# Human Induced Pluripotent Stem Cell-Based Modelling of Spinocerebellar Ataxias

**DOI:** 10.1007/s12015-021-10184-0

**Published:** 2021-05-25

**Authors:** Marina P. Hommersom, Ronald A. M. Buijsen, Willeke M. C. van Roon-Mom, Bart P. C. van de Warrenburg, Hans van Bokhoven

**Affiliations:** 1 Department of Human Genetics, Donders Institute for Brain, Cognition, and Behaviour, Radboud University Medical Center, 6500 HB Nijmegen, The Netherlands; 2 Department of Human Genetics, Leiden University Medical Center, 2300 RC Leiden, The Netherlands; 3 Department of Neurology, Donders Institute for Brain, Cognition and Behaviour, Radboud University Medical Center, 6500 HB Nijmegen, The Netherlands; 4 Department of Cognitive Neuroscience, Donders Institute for Brain, Cognition and Behaviour, Radboud University Medical Center, 6500 HB Nijmegen, Netherlands

**Keywords:** Ataxia, Cerebellum, Disease modelling, Induced pluripotent stem cells, Neurons, Spinocerebellar ataxia

## Abstract

**Abstract:**

Dominant spinocerebellar ataxias (SCAs) constitute a large group of phenotypically and genetically heterogeneous disorders that mainly present with dysfunction of the cerebellum as their main hallmark. Although animal and cell models have been highly instrumental for our current insight into the underlying disease mechanisms of these neurodegenerative disorders, they do not offer the full human genetic and physiological context. The advent of human induced pluripotent stem cells (hiPSCs) and protocols to differentiate these into essentially every cell type allows us to closely model SCAs in a human context. In this review, we systematically summarize recent findings from studies using hiPSC-based modelling of SCAs, and discuss what knowledge has been gained from these studies. We conclude that hiPSC-based models are a powerful tool for modelling SCAs as they contributed to new mechanistic insights and have the potential to serve the development of genetic therapies. However, the use of standardized methods and multiple clones of isogenic lines are essential to increase validity and reproducibility of the insights gained.

**Graphical Abstract:**

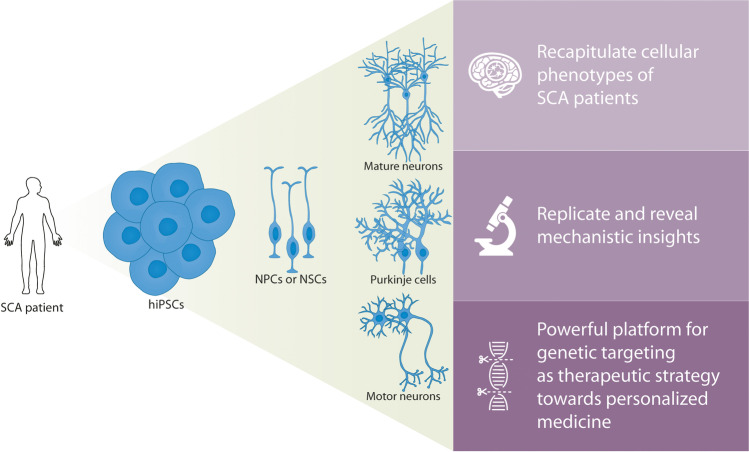

## Introduction

Autosomal dominant spinocerebellar ataxias (SCAs) are a phenotypically and genetically heterogeneous group of disorders with dysfunction and degeneration of the cerebellum and often the brainstem as their main characteristic. Today there are over 40 genes implicated in SCA [1, 2]. Seven SCAs (SCA1, SCA2, SCA3, SCA6, SCA7, SCA17 and dentatorubral-pallidoluysian atrophy) are caused by CAG repeat expansions in the coding region of a gene resulting in elongated polyglutamine (polyQ) tracts in the respective proteins. Most other SCAs are caused by conventional genetic variants (insertions, deletions, nonsense, missense or splice site variants); four SCAs are associated with intronic repeat expansions. The worldwide prevalence is estimated to be three cases per 100,000 people, with SCA3 being the most common type [2–4]. Clinically, the polyQ SCAs generally present with widespread, progressive neurological features and a reduced lifespan. Expanded CAG repeats show anticipation and within families the age of onset of SCA is negatively correlated with the repeat size [5]. In contrast, SCAs caused by conventional variants come with pure, slowly progressive cerebellar ataxia, and do not have an altered lifespan [6].

While groups of SCAs are often described by clinically distinguishable features, there is marked phenotypic overlap between the various SCAs. The clinical and still increasing genetic heterogeneity is diagnostically dealt with by next generation sequencing (NGS) platforms, which allow the simultaneous interrogation of all known SCA genes in gene panels or even entire exomes and genomes. Such large scale sequencing approaches have a high diagnostic utility. However, as a result of NGS and computational progress many variants of unknown significance in known ataxia genes and in new ataxia candidate genes are identified [7]. Consequently, there is an increasing need for functional characterization of known and candidate SCA genes and their associated genetic variants, as well as the pathophysiological pathways involved. The latter is also required to further expand our mechanistic understanding of these diseases and to design therapies. Animal models and cell models have been widely used to simulate neurodegenerative diseases, as they can be genetically manipulated relatively easily, and be subjected to a wide spectrum of well-controlled experiments. Most animal models were generated by insertion of the mutant human gene, or part thereof, into the full genome [8]. Cell models derived from humans or other species are often developed through overexpression of the gene of interest. Therefore, these models often do not recreate the full genetic or physiological context. Animals are different from humans and it has proven difficult to translate results from animal experiments into clinical application [9]. Also, for many disease-causing variants, there are no (humanized) animal models available, as it is not feasible to generate transgenic animal models for every rare disorder. Lastly, the European Union aims in their new directives to advance the development of alternative model systems to replace animal studies. The discovery that human somatic cells could be reprogrammed to human induced pluripotent stem cells (hiPSCs) has opened new opportunities to study diseases and develop therapeutic interventions [10]. Methods to differentiate hiPSCs into virtually any cell type now allows researchers to study disease-specific variants in their own genetic background. Due to the unlimited self-renewal capacity of hiPSCs and their ability to differentiate into neural lineages, we now have wide access to disease-relevant cells and tissues (organoids) that were previously largely unavailable. Moreover, with the advent of CRISPR-Cas techniques [11], it is now relatively easy to generate isogenic lines which have the exact same genetic makeup, except for the disease-causing mutation. In the last decade, SCAs have been modelled using hiPSCs, which provided us with additional insights. These hiPSCs are highly instrumental *in vitro* models for brain disorders with possibilities to combine different cell types and mimic brain regions. Nonetheless, hiPSCs and their derived models should not be regarded as a reflection of adult brain tissue but instead should be considered as reflecting relevant disease mechanisms useful to study rare disorders. For instance, by pushing the organoid differentiation towards hindbrain development, it is possible to create an hiPSC-derived cerebellar organoid [12]. This cerebellar organoid contains many different structures, but is anatomically different from the human cerebellum, and it is less predictable where certain anatomical structures will develop [13]. Still, disease-relevant changes involving different cell types and brain structures can be studied in these models.

In this review, we will focus on the research that has been conducted with SCA hiPSCs and what knowledge has been gained from these experiments, with respect to disease pathology and mechanisms, and therapeutic perspectives. We will show that SCA hiPSCs or hiPSC-derived cells (1) are appropriate models with features similar to those observed in SCA patients, (2) allow for detailed investigations on disease mechanisms in SCA, and (3) represent a critical platform towards the development of (personalized) medicine in SCA.

## HiPSC Differentiation to Neural Lineages: Protocols and Read-outs

**Fig. 1:**
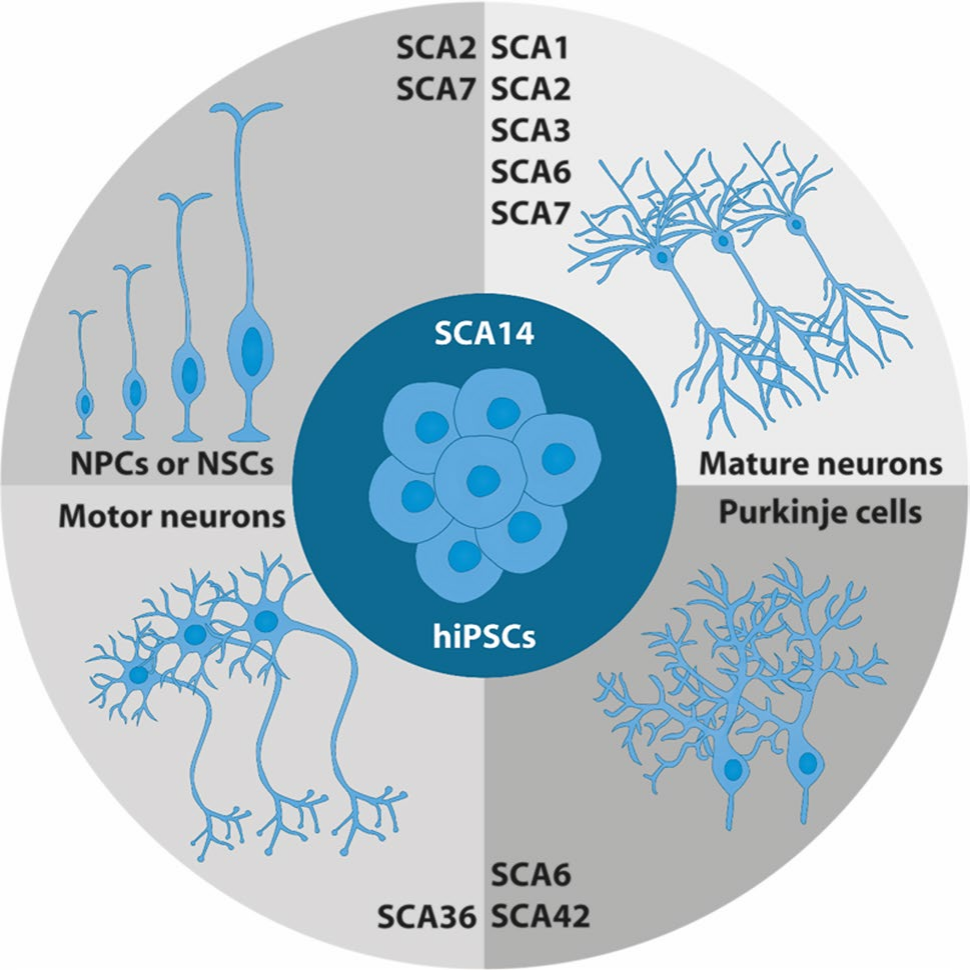
**SCA hiPSCs have mainly been differentiated to the neural lineage.** The final cell types are listed on the horizontal axis while the SCAs that have been modelled by these cell types are listed on the vertical axis. Culture-driven protocols have been used for the generation of NPCs or NSCs, mature neurons and motor neurons, yielding heterogeneous cell populations. Abbreviations: hiPSCs = human induced pluripotent stem cells; NPCs = neural progenitor cells; NSCs = neural stem cells; SCA = spinocerebellar ataxia

HiPSCs provide a powerful platform for investigating disease mechanisms and pathology, because they can be differentiated into disease-relevant cell types and tissues. Most studies so far have differentiated SCA patient-derived hiPSCs to the neural lineage (Fig. 1, Table 1). One of the greatest breakthroughs in the field of modelling SCA by hiPSCs, was the differentiation of these cells to Purkinje cells [14–19]. Purkinje cells constitute the most and universally affected neuronal population in SCAs. After 17 weeks of differentiation, hiPSC-derived Purkinje cells transcriptionally resemble late-juvenile mouse Purkinje cells, when the cerebellar circuitry has formed and dendritic arbours of Purkinje cells are almost mature [18]. Differentiation of hiPSCs into Purkinje cells is, however, laborious and time-consuming, and requires co-culturing with fetal cells or embryonic mouse cell cultures. Therefore, only a few studies investigating SCA in hiPSC-derived Purkinje cells have been reported (Fig. 1, Table 1).

**Table 1:** Disease pathology in SCA patient-derived cells

Disease	hiPSC clones	Cell Type	Additional triggers	Pathology read-outs	Main phenotype	Relevance according to criteria [38]			Reference
						Cell type	Trigger	Read-outs	
PolyQ SCAs									
SCA1	3 patients 3 controls	Neurons (culture-driven)	NA	Gene expression	NA	++	NA	NA	[44]
SCA1	1 patient	Neurons (culture-driven)	NA	NA	NA	++	NA	NA	[153]
SCA2	1 patient 1 control	NSCs (culture-driven)	NA	Differentiation, cell viability, immunostaining, Western blot	Abnormal formation of neuronal rosettes	+	NA	+	[54]
SCA2	1 patient 1 control [54]	NSCs (culture-driven)	NA	Gene expression	Expression of antisense (expanded) *ATXN2*	+	NA	NA	[69]
SCA2/3	2 x 2* SCA2 patients 2 x 2 SCA3 patients 1 x 3 controls	Neurons (culture-driven)	Glutamate	Cell viability, 1C2 immunostaining, mitochondrial morphology and function	PolyQ aggregates and distorted mitochondrial microstructures	++	±	++ [74, 154]	[66]
SCA3	2 x 4 patients 2 x 2 controls	Neurons (culture-driven)	Glutamate	Western blot, calcium imaging, electrophysiology	Glutamate induced ATXN3 aggregates	++	±	++ [82, 155, 156]	[74]
SCA3	2 patients 3 controls	Neurons (culture-driven)	Glutamate	Gene expression, immunostaining, Western blot, calcium imaging	No glutamate induced ATXN3 aggregates	++	±	++ [74]	[76]
SCA3	2 x 1 patient 2 controls (1 x hESC)	Neurons (culture-driven)	NA	Immunostaining, Western blot	Impaired protein degradation during differentiation	++	NA	NA	[21]
SCA3	3 patients 4 controls	Neurons (culture-driven)	Ionomycin	Western blot	Cleavage of ATXN3 by calpains	++	NA	NA	[80]
SCA3	3 patients 2 controls [74]	Neurons (culture-driven)	NA	NA	NA	++	NA	NA	[83]
SCA3	1 patient 1 control	Neurons (culture-driven)	Ionomycin	Western blot	Cleavage of ATXN3 by calpains	++	NA	NA	[79]
SCA3	1 patient 1 control 2 x 1 isogenic control (corrected patient)	Neurons (culture-driven)	Glutamate	Immunostaining, Western blot	Normal neuronal differentiation when expansion is deleted	++	±	NA	[22]
SCA3	2 patients 2 controls	Neurons (culture-driven)	NA	Cell viability, immunostaining, filter trap assay	PolyQ aggregates	++	NA	++ [74]	[73]
SCA3	1 control	Neurons (culture-driven)	NA	Gene expression	NA	++	NA	NA	[122]
SCA3	? patients	Neurons (culture-driven)	NA	Gene expression, immunostaining	NA	++	NA	NA	[157]
SCA3	3 x 3 patients 3 x 1 control 6 HD patients 5 controls (1 x hESC)	Neurons and NSCs (culture-driven)	Glutamate	Morphology, immunostaining, Western blot, calcium imaging electrophysiology	Glutamate induced ATXN3 aggregates in neurons, but not in NSCs	++	±	++ [74, 82, 155, 156]	[75]
SCA6	3 x 3 patients 1 x 4 controls	Purkinje cells	T3 depletion	Cell viability, morphology, immunostaining	Increased levels of Ca_v_2.1 but decreased levels of α1ACT	++	±	+	[14]
SCA6	3 x 2 patients 3 x 1 control	Neurons (culture-driven)	Glutamate	Cell viability, immunostaining, electrophysiology	Normal expression and function of Ca_v_2.1	+	±	+	[85]
SCA7	3 x 1 patient 1 hESC control	Neurons (culture-driven)	NA	Immunostaining	NA	++	NA	NA	[99]
SCA7	6 – 8 x 3 patients 2 – 8 x 2 controls 1 isogenic control (corrected patient)	NPCs (culture-driven)	NA	Cell viability, Western blot, mitochondrial morphology and function	Reduced cell survival, aggregation of ATXN7 and impaired oxidative metabolism	+	NA	++ [100]	[100]
Intronic repeat expansion SCAs									
SCA36	3 patients 3 controls	Motor neurons (culture-driven)	NA	Differentiation, gene expression, FISH	Intranuclear RNA foci	++ [158]	NA	++ [159]	[25]
Conventional mutation SCAs									
SCA14	2 x 4 patients 1 x 2 controls	hiPSCs	Activation of PKCγ by PMA	Gene expression, immunostaining, Western blot	Cytoplasmic mislocalization of PKCγ aggregates	-	±	++ [101]	[101]
SCA42	1 patient 1 control	Purkinje cells	NA	Morphology and immunostaining	Differentiation not affected	++	NA	+	[15]

++ = highly relevant, + = relevant, ± = unknown, - = irrelevant

*NA* not applicable, *FISH* fluorescent in situ hybridization, *hESC* human embryonic stem cell, *hiPSC* human induced pluripotent stem cell,*NPCs* neural progenitor cells, *NSCs* neural stem cells, *PMA* phorbol 12-myristate 13-acetate, *SCA* spinocerebellar ataxia

*n x *m* stands for *n* clones per *m* patients or control subjects

The majority of studies took the approach of differentiating SCA hiPSCs to cortical cells, for which there are two ways: culture-driven differentiation and transcription factor-driven forced differentiation. Today, various culture-driven protocols have been published in which the embryonic differentiation is more or less recapitulated by either formation of embryoid bodies with neuronal rosettes generating neural progenitor cells (NPCs) or by continuously differentiating hiPSCs to mature neurons [20]. These protocols require a thorough characterization of the cell types present at each developmental stage to confirm sufficient efficiency in obtaining the desired cell type. In contrast, by using differentiation protocols with forced overexpression of transcription factors, generally more homogeneous and mature populations of cell types are generated [20]. However, the latter has so far not been used for differentiating SCA hiPSCs (Table 1). When comparing the culture-driven differentiation protocols used for SCA hiPSCs, subtle differences can be found. This not only hampers a critical reflection on the reproducibility of results, but also makes it difficult to compare data for the different SCAs.

Overall, SCA hiPSCs seem to differentiate well to neural cells. Also, in polyQ SCAs, the repeat seems to remain stable during the whole process of reprogramming, passage and differentiation [21, 22]. After differentiation to the final cell type (Fig. 1), several read-outs can be used on SCA hiPSC-derived cells. These read-outs generally focus first on the phenotype of the hiPSC-derived cells. Usually this starts with investigating the survival and morphology of the cells, or the expression and distribution of the gene and protein of interest. In polyQ SCAs, cells are often stained with an antibody named 1C2, which was developed to target TATA-box-binding protein (TBP), but also emerged to bind specifically to expanded polyQ tracts [23]. Moreover, protein aggregates are often detected for the respective proteins of the polyQ SCAs [24]. For the intronic repeat expansion SCAs, SCA3 and SCA8, RNA foci can be detected by fluorescence in situ hybridization (FISH) using repeat-specific oligonucleotides [25–28]. Most of these read-outs are also used for human post-mortem brain tissues. The phenotype of hiPSC-derived cells – when investigated with these tools – can thus be considered as reflective of relevant disease pathology.

## Disease Pathology and Mechanisms

When modelling SCAs, it is useful to first study the disease pathology of the particular subtype in humans by using structural MRI scans and neuropathological investigations of post-mortem brain tissue, preferably at various disease stages. Over the years, post-mortem brain and MRI studies have revealed that SCAs are primarily characterized by atrophy of the cerebellum and brainstem [29, 30]. However, the amount of neuronal loss in the cerebellum and other affected brain areas varies greatly between the different SCAs [6, 31, 32]. Given the genetic heterogeneity of SCAs, numerous pathways seem to be involved in the pathogenesis. However, it has been shown that ataxia genes may eventually converge to two modules that affect neuronal homeostasis [33]: 1) calcium homeostasis and neuronal signalling [34, 35], and 2) cellular stress response [33, 36]. In most polyQ SCAs, a dysregulation of the transcriptional machinery is a more prominent feature [36, 37].

So far, hiPSC-based cell models have been established and studied for many SCAs (Table 1), but not all studies reported standard read-outs on disease pathology as discussed above. In this section, we will focus on which disease pathologies and mechanisms have been confirmed in SCA hiPSCs and which new knowledge was gained from these models. We will discuss studies that have reported such standard read-outs on different SCA hiPSC-derived cells in detail. To correlate the observed disease pathologies with existing human data, we will finish with an evaluation of strengths and weaknesses according to three criteria that were laid down by Vincent et al. in 2015 [38]. These criteria were proposed to increase the reproductivity of phenotypic assays (compound screening systems) in the pharmaceutical industry. First of all, the assay system should be relevant for the disease by using a cellular system with endogenous expression of the mutant protein(s) of interest, such as patient-derived hiPSCs. Secondly, the trigger of the disease phenotype needs careful consideration. In patient-derived hiPSCs, the genetic alterations present in the genome of the patient can be considered as the disease trigger. Last, the read-out should have clinical relevance, for example genetic or protein biomarkers and cell physiological hallmarks of the disease [38].

### SCA1 and ATXN1

Normally, ATXN1 mainly localizes to the cell nucleus and is involved in transcription regulation and RNA splicing [39–41]. It interacts with several transcription factors and RNA binding proteins in large protein complexes, including the transcriptional repressor Capicua (CIC) [42] and splicing factor RBM17 [43]. The phosphorylation of Ser776 is necessary for the interaction of ATXN1 with RBM17 [43]. This interaction was enhanced when ATXN1 carries an expanded polyQ tract. This observation supports the hypothesis that ATXN1-CIC and -RBM17 complexes are in a dynamic equilibrium, which is disturbed by expansion of ATXN1 [43]. However, cerebellar phenotypes were absent when ATXN1-CIC complex formation was completely abolished by mutating two crucial residues in ATXN1 in transgenic mice carrying 82 CAG repeats [44]. This suggests that the interaction between expanded ATXN1 and CIC acts in a gain-of-function manner. Given the transcriptional regulatory function of this complex, Rousseaux et al. investigated the transcriptional profile in SCA1 hiPSC-derived neurons and found that genes involved in glutamatergic neurotransmission were downregulated [44]. Interestingly, SCA1 transgenic mouse models show defects in glutamatergic neurotransmission [45, 46]. This links the dysregulation of the transcriptional machinery to a defect in neuronal signalling in SCA1. Hitherto, no standard read-outs for disease pathology have been performed for SCA1 hiPSC models. This may affect the reproducibility of gain-of-function of expanded ATXN1 and CIC and defects in glutamatergic signalling in these hiPSC-derived models.

### SCA2 and ATXN2

ATXN2 localizes to polyribosomes and the Golgi apparatus and interacts directly and indirectly with RNA [47–51]. Therefore, it is believed ATXN2 plays an important role in transcriptional regulation. How expanded ATXN2 leads to a SCA2 phenotype is still under debate, but cell and animal studies support a gain-of-function mechanism [48, 52, 53].

While most studies did not report any difficulties differentiating SCA hiPSCs, one study reported such difficulties in differentiating SCA2 hiPSCs to neural stem cells (NSCs) [54]. SCA2 hiPSCs formed abnormal neural rosettes, in which the cells grew in a cyst-like structure with NSCs emerging from the structure. Also, SCA2 NSCs tended to spontaneously differentiate at early passages, expressed less ATXN2, and had a shorter lifespan than control NSCs [54]. The observed differentiation defects of SCA2 NSCs might be relevant for the neurodevelopmental features of the disorder [55–57]. Although some SCAs, like SCA1, SCA10, SCA13 and SCA17, show features of neurodevelopmental disorders such as epilepsy and intellectual disability [29, 58, 59], only extreme CAG repeat expansions cause such manifestations in SCA2 [60–65]. As Xia et al. only reprogrammed and differentiated one SCA2 hiPSC line, this phenotype might have been specific for this patient with 20/44 CAG repeats. Indeed, Chuang et al. reported successful differentiation of hiPSCs derived from two SCA2 and two SCA3 patients to neurons [66]. SCA neurons showed polyQ-1C2 positive punctate and distorted mitochondrial microstructures. Upon addition of glutamate, SCA neurons showed mitochondrial bioenergetic failure and cell viability decreased significantly in a time-dependent manner.

As natural antisense transcripts have been described to contribute to disease pathogenesis in SCA7 [67] and SCA8 [68], Li et al. explored the expression of antisense transcripts of *ATXN2* [69]. In SCA2 hiPSC-derived NSCs, both the normal and expanded antisense transcripts were present and by sequestering MBNL1 to CUG RNA foci, antisense transcripts of expanded *ATXN2* triggered mis-splicing of MBNL1 target genes in SCA2 NSCs [69]. This new mechanism for SCA2 might play an important role in the disease pathogenesis.

### SCA3 and ATXN3

The ubiquitously expressed ATXN3 protein binds to polyubiquitylated proteins in the cytoplasm and acts as a deubiquitinating enzyme [70, 71]. Expanded ATXN3 localizes more readily to the nucleus, forming intranuclear aggregates that contribute to neuropathology [72]. The expanded protein is still able to bind ubiquitin in hiPSC-derived neurons [22] and shows normal proteasome activity, with increased sensitivity for proteasome inhibition [73]. This hinted towards toxic gain-of-function rather than a loss-of-function of the protein.

The first study to generate and investigate SCA phenotypes in patient-derived hiPSCs was described in 2011 [74]. Koch et al. generated hiPSC-lines from four SCA3 patients with CAG repeat expansions in exon 10 of *ATXN3* and differentiated these hiPSCs to neurons. Although no neurophysiological differences were observed between SCA3 and control neurons under naïve conditions, glutamate stimulation led to Ca^2+^-influx and Ca^2+^-dependent cleavage and subsequent aggregation of fragments of expanded ATXN3. This aggregation was limited to SCA3 neurons and was not observed in SCA3 fibroblasts or hiPSCs. Furthermore, the aggregates were positively stained for both polyQ-1C2 and TBP [74]. Recently, these phenotypes have been replicated by Thiruvalluvan et al. [75], who additionally showed that aggregates were not formed in NSCs challenged with glutamate. Also, Chen et al. showed, by a filter trap assay, that unstimulated SCA3 neurons contained 1C2-positive insoluble protein, while cell viability was not affected [73]. However, others have not been able to reproduce these phenotypes [22, 76]. While it was suggested that different reprogramming and neuronal differentiation protocols may yield different neuronal populations that are better at clearing cleaved and aggregated ATXN3, these discrepancies do illustrate the difficulties encountered in phenotypic assays using hiPSC-derived models. As Thiruvalluvan et al. showed in their study, the protein homeostasis network is heavily modulated during differentiation, with high expression of the anti-amyloidogenic chaperone DNAJB6 during the stem cell stage, whereas a lower expression is observed in mature neurons [75]. This suggests that hiPSCs are intrinsically protected against protein aggregation and explains why neurons are more prone to aberrant protein aggregation. Different neuronal differentiation protocols might therefore influence the ability of neurons to clear protein aggregates.

Over the last decade, the role of ATXN3 cleavage by enzymes has been investigated in hiPSC-derived cells as an important molecular mechanism in SCA3. In SCA3 models, cleavage of ATXN3 was shown to be mediated by both caspases and calpains [77, 78]. In hiPSC-derived neurons, it has now been confirmed that ATXN3 aggregate formation only involved cleavage by Ca^2+^-dependent calpains [74, 76, 79, 80]. SCA3 hiPSC-derived models showed increased calcium levels [66], activating calpains to produce ATXN3 fragments that are more prone to aggregate in the nucleus [81]. Calcium dyshomeostasis in these models was also linked to an altered expression of glutamate receptor subunits, leading to changes in receptor subunit composition [66]. This provides evidence for affected neuronal signalling in SCA3. Some studies now also point towards distorted protein quality control by molecular chaperones. Since DNAJB1 co-localized with ATXN3 aggregates [82], Evert et al. looked at the expression of this co-chaperone and found decreased levels in SCA3 neurons, whereas levels of two miRNAs regulating DNAJB1 were increased [83]. Additionally, another member of the HSP40 protein family, DNAJB6, shows strongly reduced expression when hiPSCs are differentiated to mature neurons [75]. Expanded ATXN3 was not able to form aggregates in highly proliferative cells with high DNAJB6 expression, but neurons with decreased DNAJB6 expression showed vulnerability to ATXN3 aggregate formation. This suggests that a dysregulation of protein homeostasis is a key mechanism in SCA3 that may not be directly mediated by expanded ATXN3, but rather by its cleavage products or interactors.

### SCA6 and Cav2.1/α1ACT

The pore-forming subunit α1A of the P/Q-type voltage-gated calcium channel Ca_v_2.1 localizes to presynaptic terminals of central synapses [84]. The effect of the polyQ expansion in its C-terminal tail on the function of the channel still remains elusive, as studies have yielded conflicted findings [85–91]. The discovery of an internal ribosomal entry site in *CACNA1A* mRNA and the second product α1ACT, functioning as a transcription factor [92], however, linked the molecular mechanism behind SCA6 to transcriptional dysregulation.

For SCA6, two independent studies have utilized different models to study disease pathology and mechanisms. Ishida et al. differentiated hiPSCs from three SCA6 patients and two healthy donors to mature Purkinje cells and observed no differences in frequency of cells, dendritic field area, total length of dendrites, or soma diameter [14]. As α1ACT also carries the expanded polyQ tract, Ishida et al. used different antibodies to look at the levels of both Ca_v_2.1 and α1ACT [14]. While Ca_v_2.1 protein levels increased in a gene dosage-dependent manner, α1ACT protein levels and gene expression of its targets *TAF1* and *BTG1*, which are relevant for neuronal survival [92], decreased in correlation with gene dosage. Also, expanded α1ACT remained in the cytoplasm, due to hampered transport to the nucleus [14]. After depletion of thyroid hormone T3, which supports maturation and maintenance of Purkinje cells, Ishida et al. found that SCA6 Purkinje cells showed decreased cell viability and dendritic field area [14]. The second SCA6 study was that of Bavassano et al., who differentiated two patient-derived hiPSC lines to neuronal cells, containing both glutamatergic and GABAergic neurons [85]. In contrast to the findings of Ishida et al., no differences in Ca_v_2.1 and α1ACT expression and distribution were found, but they found lower expression levels of α1ACT target *GRN*. This supports the hypothesis that polyQ expansion in α1ACT affects the transcriptional machinery. Furthermore, electrophysiological characteristics were comparable to control neurons. When the neuronal cultures were stressed with glutamate, SCA6 neurons showed a reduced cell viability compared to treated control neurons [85]. A reason for these discrepant findings could be the difference in cell types used. Purkinje cells are cerebellar inhibitory neurons that have a very different developmental trajectory and expression profile compared to cortical glutamatergic neurons [93]. Purkinje cells are characterized by high levels of Ca_v_2.1 [94], whereas cortical glutamatergic neurons also express N-type, L-type or T-type voltage-gated calcium channels [95]. Another reason could be that the neurons had not reached the stage of maturation to show a phenotype. In 5-week-old immature Purkinje cells, Ishida et al. did not detect any differences in expression levels of Ca_v_2.1 and α1ACT [14], which might be the same for 5-week-old neurons in the Bavassano study. This would imply that the maturity of the culture and the possibility of increased toxicity of aggregates in older cultures has to be taken into account when modelling SCAs with hiPSCs.

### SCA7 and ATXN7

ATXN7 is a component of two transcriptional coactivator complexes with histone acetyltransferase activity, SPT3/TAF9/GCN5 acetyltransferase complex (STAGA) and TBP-free TAF complex (TFTC) [96, 97], which possess a subcomplex with ubiquitin protease UPS22 [98]. Expanded ATXN7 has been shown to act in a dominant-negative manner, inhibiting histone acetylation [97].

In 2012, Luo et al. showed for the first time that SCA7 patient-derived hiPSCs can be generated and differentiated to the neural lineage [99]. Subsequently, Ward et al. generated hiPSCs from three SCA7 patients, and two healthy subjects, all from two independent families, and differentiated these to NPCs [100]. Though indicators for aggregation of ATXN7 were weak and similar between all SCA7 and control NPCs, one of the patient NPCs showed decreased survival. Based on the clinical overlap between SCA7 and mitochondrial disorders, the authors studied the mitochondrial network length and noticed a decrease in the SCA7 patient with the largest repeat. To create isogenic lines for direct comparison, they knocked out endogenous *ATXN7* in one of the SCA7 hiPSCs by CRISPR/Cas genome editing and reintroduced *ATXN7* with either 10 or 113 CAG repeats by a lentiviral vector. After differentiation, NPCs carrying 113 CAG repeats in *ATXN7* showed markedly increased accumulation of aggregated ATXN7 and cell death. These cells also displayed a reduced oxygen consumption rate and increased extracellular acidification rate [100]. This study shows the strength of using isogenic cell lines in phenotypic assays. In these isogenic lines, Ward et al. also investigated NAD^+^ pathways as the key element in oxidative phosphorylation in SCA7 hiPSC-derived NPCs [100]. SCA7 NPCs showed lower levels of NAD^+^ caused by an imbalance in the tryptophan/kynurenine pathway for *de novo* synthesis of NAD^+^. These findings again show how a defect in the transcriptional machinery can be linked to affected cell homeostasis.

### SCA14 and PKCγ

The conventional protein kinase PKCγ plays a central role in second messenger signalling. To date, 40 different genetic variants have been reported in *PRKCG* causing SCA14 [101]. Most of these variants occur in the C1 and C2 regulatory domains of the protein, which bind diacylglycerol and Ca^2+^, respectively. Wong et al. derived hiPSCs from four SCA14 patients [101]. Two of these patients were carrying a variant in the *PRKCG* gene predicting a His36Arg substitution and the other two patients had a variant predicting a His101Gln substitution in the encoded PKCγ protein. Although *PRKCG* is generally known to be expressed in the central nervous system, PKCγ solely localizes to neurons, and shows a high abundancy in Purkinje cells [101–104], Wong et al. found a marked expression of *PRKCG* in control and SCA14 hiPSCs [101]. Control hiPSCs showed small PKCγ puncta in the cytoplasm, whereas large cytoplasmic aggregates were observed in SCA14 hiPSCs, similar to their observations in post-mortem brain tissue. When hiPSCs were stimulated with a PKC activator, the aggregation of PKCγ was further enhanced in SCA14 hiPSCs. Whereas PKCγ translocated to the plasma membrane in control hiPSCs upon activation, it remained aggregated in the cytoplasm of SCA14 hiPSCs, impairing autophagy of aggregates and increasing PKCγ autophosphorylation and downstream signalling. The aggregates did not show co-localization with ubiquitin or p62, consistent to observations in post-mortem brain tissue [101]. This suggests that these SCA14 variants lead to a combined loss-of-function of PKCγ at the plasma membrane and a gain-of-function of the protein at the cytoplasmic level. This study also confirmed previous observations of increased PKCγ activity [105, 106], while others reported cytoplasmic PKCγ aggregates [107, 108] and affected diacylglycerol binding by the C1 domain [106].

### SCA36 and Nucleolar Protein 56

SCA36 is an intronic repeat expansion SCA caused by a GGCCTG hexanucleotide repeat expansion in *NOP56*. *NOP56* encodes nucleolar protein 56, which is involved in the biogenesis of the 60S ribosomal subunit [109]. The pathogenic mechanism of the intronic hexanucleotide repeat is still not fully understood, but may involve loss-of-function of nucleolar protein 56 or toxic RNA gain-of-function by intranuclear aggregation to RNA foci, as suggested for other intronic repeat expansion disorders [110, 111]. Matsuzono et al. have derived hiPSCs from three SCA36 patients and three healthy subjects and differentiated these to motor neurons [25]. No differences were found in neuronal differentiation. However, both SCA36 hiPSCs and neurons showed a significant increase in intranuclear RNA foci as detected by FISH and a significant decrease in expression of *NOP56 *[25]. These phenotypes suggest that both loss-of-function and toxic RNA gain-of-function might be involved in the pathogenic mechanisms of SCA36, but a thorough characterization of downstream effects is still needed.

### SCA42 and Cav3.1

SCA42 is caused by conventional mutations in the *CACNA1G* gene, which encodes the pore-forming subunit of the T-type voltage-gated calcium channel Ca_v_3.1. Morino et al. derived hiPSCs from one patient with a predicted Arg1715His substitution in Ca_v_3.1 and one healthy subject and differentiated these to Purkinje cells [15]. No obvious differences were observed in morphology and expression of the Purkinje cell-specific marker L7. Future studies need to clarify possible disease pathologies and mechanisms involved that would cause a SCA42 phenotype.

When evaluating the abovementioned studies on strengths and weaknesses (Table 1), we can conclude that most studies utilized relevant cell types, triggers and read-outs. Reasons for not meeting the full “Vincent criteria” were mostly the suboptimal cell type, i.e. not the cell type most affected by the disease, and lack of read-outs that better reflect human pathology. In several polyQ SCAs, widespread neuronal loss is observed [29], whereas in SCA6 loss of cerebellar Purkinje cells is more pronounced [29, 112]. Therefore, SCA6 is ideally modelled by Purkinje cells, while the use of cortical neurons is justified in other polyQ SCAs. Apart from extreme CAG expansions [60–65, 113], the polyQ SCAs have not yet been correlated with developmental issues and can be considered as neurodegenerative diseases of the central nervous system. Although neurodegenerative diseases can have a developmental component [114], the next step in the field of polyQ SCAs might be to differentiate hiPSCs to neurons by forced overexpression of transcription factors to generate a more homogeneous population of more mature neurons. These neurons might be able to capture the neurodegenerative nature of polyQ SCAs. As conventional mutation SCAs normally show more isolated cerebellar pathology [6], these disorders are ideally modelled by Purkinje cells. In terms of triggering disease phenotypes in these models, all studies automatically showed relevance by using patient-derived cells carrying the relevant genetic variants. In addition to endogenous genetic variants, differences in chemical compositions of culture medium were applied. Higher concentrations of glutamate were applied to SCA2, SCA3 and SCA6 neurons to monitor the effect of excitation-induced calcium influx in SCA neurons [74]. SCA6 Purkinje cells were deprived of T3 to affect the maintenance of these cells [14]. Currently, it is not clear whether the addition of glutamate to the medium or T3 depletion is relevant for human pathology. For example, neurometabolic profiling in SCA3 patients revealed decreased, rather than increased, levels of glutamate [115]. Yet, it was suggested that this decrease of glutamate reflected neuronal loss and dysfunction in these patients, so elevated levels of glutamate might still have served as an initial trigger in SCA3 patients. Also for SCA6 it is not clear whether the addition of glutamate to the medium is relevant for human pathology [116, 117] and reduced levels of T3 have never been described in patients. It is therefore difficult to assess the relevance of these triggers. Concerning read-outs, various types have been applied, but not all read-outs are consistently applied. To fully match human pathology, read-outs should at least encompass cell viability, morphology, gene or protein expression, and distribution of the gene of interest and co-localization with specific markers for the disease. For polyQ disorders, these markers are aggregates that can be visualized by 1C2, whereas RNA foci are specific markers for intronic repeat expansion SCAs, SCA3 and SCA8.

To conclude, hiPSC-based cell models have been established and studied mainly for the polyQ SCAs with a focus on SCA3. HiPSCs and hiPSC-derived cell models allow for detailed investigations of disease mechanisms. These models do recapitulate pathological phenotypes that also have been observed in patients, faithfully reproduce previous mechanistic findings in other model systems, but also provide additional insights by using disease-relevant human cells. Still, phenotypic read-outs need to be improved and consistently applied across studies to eventually gain robust insights into the mechanisms involved. On top of that, the maturity of the cultures as well as the use of isogenic cell lines have to be considered when modelling SCAs with hiPSC-derived cells.

## Therapeutic Perspectives

Knowledge about molecular mechanisms of disease point to avenues to design and test therapeutic approaches. For SCAs, no disease-modifying treatments exist, but some drugs are applied for symptomatic relief. Riluzole is one of the medications with a proven benefit to reduce ataxia symptoms [118–120]. It is a modulator of calcium-activated small conductance potassium (SK) channels [121], reducing neurotransmission. In fact, riluzole was the only drug that alleviated cell survival, oxygen metabolism, and calcium homeostasis in SCA2, SCA3 and SCA6 hiPSC-based models [14, 66]. Other drugs, such as D-APV (NMDA-R antagonist), NBQX (AMPA-R antagonist) and Tetrodotoxin (sodium channel blocker), were able to relief the cells from aggregates in SCA3, but did not have any follow-up investigations [74]. In addition to drugs that target neuronal signalling in SCA, compounds stimulating autophagy or proteasome activity, such as rapamycin and Chinese herbal medicine extract NH037, exhibited an increased breakdown of expanded ATXN3 and a reduction of cytotoxicity and oxidative stress in SCA3 hiPSC-derived neurons [21, 73]. As these compounds target the pathways involved in the cellular stress response, follow-up studies could clarify whether these are indeed promising candidates for clinical trials. Next to pharmacological treatments, genetic targeting seems a very promising therapeutic approach. For polyQ and intronic repeat expansion SCAs, post-transcriptional gene silencing strategies have used virus-mediated delivery of artificial microRNAs [122] or antisense oligonucleotides (AONs) [25, 123]. Especially AONs bear great therapeutic potential as they can be disease- and allele-specific, are relatively easy to administer, and are efficiently taken up by brain cells with a stability for months [124]. Hitherto, only SCA36 hiPSC-derived motor neurons have been treated with AONs, but several animal and cell models for SCA1, SCA2, SCA3 and SCA7 have been highly instrumental to test different AONs with variable success rates [125–134]. Moreover, the rapid development of genome editing strategies by CRISPR/Cas makes it possible to correct causative genetic variation, which is of ultimate importance in personalized medicine. Next to the knock-out of *ATXN7* described above [100], only one other study has used this technology as a first step towards these aims. Ouyang et al. removed CAG repeats in SCA3 patient-derived hiPSCs by using two different guide RNAs flanking the repeat [22]. Corrected hiPSCs expressed a normal and a truncated ATXN3 and were still able to differentiate to neurons. No off-target effects were observed. Although the strategy of Ward et al. to introduce normal ATXN7 with lentivirus in their CRISPR/Cas-mediated knock-out lines is biologically more relevant, the genome editing strategy of Ouyang et al. might be therapeutically more feasible, because of the theoretical potential of insertional mutagenesis using lentiviral vectors [135]. The improvements in this field might eventually lead to generation of hiPSCs with two alleles with repeats within the normal range for the repeat expansion SCAs and mutation correction for conventional mutation SCAs. However, challenges regarding delivery and specificity have probably contributed to a lack of clinical trials for brain disorders using genome editing. The question remains if CRISPR/Cas genome editing will also become available for therapeutic purposes in SCAs, but the hiPSC model system can be used for experiments that test these approaches.

## Conclusions and Future Perspectives

The key to understanding the pathophysiology of SCAs and developing disease-modifying therapies is the establishment of disease-relevant models. As hiPSCs carry the genetic makeup of the patients, they provide a powerful tool for modelling this genetically heterogeneous group of disorders. Over the past decade, hiPSC technology has evolved to one of the basic research tools to model diseases. The studies reviewed here have shown that hiPSC-based models faithfully recapitulate cellular phenotypes observed in SCA patients. Moreover, it is very promising that most studies have been able to replicate previously observed findings in animal and cell models, while in some studies new mechanistic insights were obtained. Most of the mechanisms involve affected neuronal signalling or a defect in the cellular response to stress, making these pathways important therapeutic targets. In the last few years, genetic targeting is up-and-coming as a new therapeutic strategy and hiPSCs provide a powerful platform for these techniques and thus for personalized medicine. However, certain opportunities remain to be exploited and challenges to be dealt with in the future. SCA hiPSC studies have mainly focused on polyQ SCAs, but other types remain largely unexplored. Furthermore, with the ability to differentiate hiPSCs to all the main cell types in the brain and the possibility of co-culturing, we are now able to reconstitute key interactions between neuronal cells and to generate brain organoids. The advantage of organoids is the spatial organization of the different cell types, which allows researchers to understand cell type-specific disease mechanisms. This creates the opportunity to model affected neuronal signalling pathways in SCA even more closely. Moreover, stem cell-based replacement therapies have been studied with embryonic, neural and mesenchymal stem cells in SCA1, SCA2 and SCA3 mouse models (as reviewed in [123]), but to date no hiPSC-based replacement studies have been reported in the SCA field. A persisting challenge in hiPSC technology is the high level of interindividual variability, especially in the clinically and genetically heterogeneous group of SCA patients. A single hiPSC model will not be able to recapitulate the different aspects of pathophysiology in all SCA patients. Nowadays, many new hiPSC lines carrying different genetic variants of SCAs have been generated and registered [136–149], which makes it easier to utilize multiple clones from multiple patients. Among these lines, also isogenic control lines created by CRISPR/Cas are listed [150–152], which can serve as proper controls that take genetic background into account as an important variable. It is therefore always important to use multiple patient and control lines and clones and, if possible, isogenic lines. Modelling SCAs within the patient’s genetic background and a human context is the strength of hiPSCs that made them revolutionize the field over the last decade, which will continue in the coming years.
